# The competence paradox: negotiating ease, risk, and creative identity in text-to-image generative AI use among art and design students

**DOI:** 10.3389/fpsyg.2026.1858187

**Published:** 2026-07-02

**Authors:** Yihan Liu, Meng Meng, Yue Zhang

**Affiliations:** 1 College of Architecture and Art, Hefei University of Technology, Hefei, China; 2 Research Center for Green Design and Low-carbon Intelligent Manufacturing, Hefei University of Technology, Hefei, China

**Keywords:** art and design, generative AI, higher education, text-to-image, UTAUT

## Abstract

**Introduction:**

The rapid diffusion of text-to-image (T2I) generative AI has intensified pressures surrounding assessment and skill reconfiguration in art and design education. However, existing research on T2I adoption in studio-based pedagogy remains limited. This study examines technology acceptance from both educators' and students' perspectives, and illustrates the transformation of intentions and actions in creative learning situations.

**Methods:**

A modified exploratory sequential mixed-methods design with an explanatory qualitative follow-up phase (QUAL-QUAN-qual) was employed. Instructor focus groups were first conducted to identify key constructs and inform the development of a contextualized technology acceptance framework. This was followed by a questionnaire survey of 417 college students, and semi-structured interviews to explain unexpected quantitative results.

**Results:**

The results indicate that performance expectancy, social influence, novelty value, and creative competence positively influence behavioral intention. In contrast, the negative effects of effort expectancy and facilitating conditions can be interpreted in light of students' shortcut-oriented use of T2I tools in coursework. Furthermore, students with different levels of competence perceive distinct risks across task stages, which helps explain the lack of significant translation from intention and creative competence into use behavior.

**Discussion:**

The findings highlight a paradox: although creative competence positively supports behavioral intention, it may also lead to more selective or restrained engagement in actual use. Accordingly, the study extends technology acceptance models in creative education by showing that T2I adoption cannot be understood solely through conventional utilitarian predictors. Instead, it is also shaped by students' interpretations of risk and their developing creative identity, particularly in authorship, originality, and skill preservation. The results reconceptualize T2I adoption as a dynamic process of negotiation between diverse student profiles and technological evolution, ultimately providing an evidence-based foundation and practical recommendations for AI pedagogy in creative education.

## 1 Introduction

Text-to-image (T2I) has become one of the most prominent and rapidly maturing branches of AI-generated content, which generates images from natural-language prompts through diffusion-based and related generative architectures (Zhang et al., 2023), with recent advances markedly improving the quality, accessibility, and controllability of image synthesis (Benbya et al., 2024; Rombach et al., 2022). The rapid evolution has accelerated the re-shaping of traditional processes of art and design production and heightened concerns about skill reconfiguration and the substitution of creative headcount in the design profession. According to the Figma (2026) report, the AI-driven democratization of design is blurring traditional role boundaries in product development, compelling designers to transition from basic execution to higher-order strategic thinking. This, in turn, has prompted art and design college education to critically reflect on rapidly evolving technologies and the ever-changing demands of the industry (Amankwah-Amoah et al., 2024; Fleischmann, 2024).

Given the distinctive nature of creative education, learning outcomes are inextricably linked to visual expression, strict norms of originality, and an iterative studio workflow in which ideas evolve through a continuous cycle of creation, critique, and refinement (Hetland et al., 2013). The T2I GenAI tool can be introduced at any stage of the process (Ringvold et al., 2024). Imagine a student making a beautiful rendering in seconds based on some text prompts: the assessment validity would be immediately questioned (Moorhouse et al., 2023; Luo, 2024).

Moreover, art and design education is not limited to the acquisition of technical skills but also involves the formation of creative identity, particularly regarding authorship, originality, and creative responsibility (Orr and Shreeve, 2017; Trede et al., 2012). The emergence of T2I GenAI also challenges students' understanding of originality and the legitimacy of their work, placing them between efficiency-oriented use and the pursuit of creative authenticity (McCormack et al., 2019). Thus, understanding how motivational and contextual factors translate into behavioral intention and actual use is crucial for this fast-evolving educational landscape. Against this background, this study adopts a three-phase sequential mixed-methods design that integrates educator-informed model building, student-based testing, and student-centered explanation. The integrated findings provide context-sensitive pedagogical observations, explanations, and implications to fill a critical gap in the literature on the application of GenAI in creative disciplines and higher education.

## 2 Literature review and overall research design

### 2.1 T2I GenAI in art and design education

T2I GenAI is both compatible with and disruptive of art and design education. Early research noted GenAI's potential for teaching aesthetics and technique (Dehouche and Dehouche, 2023), which requires visual literacy, accurate keyword use, and stronger preliminary research and trend awareness (Hwang and Wu, 2025; Medel-Vera and Gates, 2025). Thus, T2I is not simply a supporting tool; it reshapes and challenges the prompting, visualization, critique, and authorship involved in creative work and studio-based assessment (Ringvold et al., 2024; Oppenlaender, 2022; Tan and Luhrs, 2024). Recent discussions on digital labor suggest that users participate in a reconfigured form of labor, in which creative processes such as ideation, execution, and refinement are partially delegated to algorithmic systems (Fuchs, 2014). Such a transformation raises questions about the locus of creative labor and the attribution of authorship, particularly in studio-based education, where process and effort are central to assessment. GenAI is therefore increasingly judged not only in terms of what it can produce and how it is deployed, but in relation to learning, assessment, and future professional practice (Fleischmann, 2024).

Ng et al. (2021) and Chan (2023) assume that in the face of unbalanced AI literacy, instructors must weigh teaching opportunities against ethical, technological, and pedagogical considerations. Student-centered studies point out that students in art and design remain sensitive to originality, privacy, technophobia, and anxieties around AI-assisted creation (Shen et al., 2025; Li, 2025), and negotiate T2I as a learning medium as well as an element threatening future careers (Fleischmann, 2024; Wang et al., 2025). However, these studies say less about how and why students come to accept, limit, or resist them in studio-based creative education.

Methodologically, the broader higher-education literature on AI art still relies largely on short-term qualitative and case-based studies (Rong et al., 2025; Jiang et al., 2026) and single cross-sectional questionnaire surveys (Zhu et al., 2025; Xu et al., 2025; Bao and Zhang, 2026). Although more recent work has introduced interpretive, process-oriented, and predictive approaches, including neural networks (Ringvold et al., 2024; Vartiainen and Tedre, 2023; Fareed et al., 2024; Paananen et al., 2024), it still offers limited insight into the meanings students attach to their own choices and contradictions. More robust and multi-perspective research designs are therefore needed to explain how T2I GenAI acceptance unfolds in art and design education.

### 2.2 Technology acceptance and adoption models

Technology acceptance research provides the foundational theoretical framework for explaining user adoption or resistance to emerging tools. The progression from the seminal Technology Acceptance Model (TAM) to its comprehensive extensions (TAM2, UTAUT, and UTAUT2) marks a shift from basic utilitarian metrics toward multifaceted models integrating social influence, facilitating conditions, and contextual motivations (Davis, 1989; Venkatesh et al., 2003). Building on this foundation, empirical research has already examined general-purpose GenAI adoption across a range of higher-education disciplines, including language learning, business and management, and health-related education (BinJwair and Alamer, 2025; Chan and Hu, 2023; Alghasab, 2025; Elbaz et al., 2024; Sallam et al., 2023). However, the direct transfer of these conventional models to studio-based creative education is problematic. Specifically, they are less equipped to capture the distinct cognitive demands salient in T2I contexts, such as prompt crafting, output evaluation, creative control, and risk-sensitive judgment (Oppenlaender et al., 2025). To address this insufficient contextual sensitivity, recent studies have adapted and extended these models to a wider range of T2I systems (Du et al., 2023; Shen et al., 2025; Fang and Jiang, 2024; Wang et al., 2025; Li et al., 2025). Yet, these adaptations predominantly target established professionals driven by workflow efficiency or employ generic acceptance metrics. Consequently, they frequently overlook the unique vulnerabilities of design students, whose disciplinary skills, evaluative standards, and professional identities are actively forming.

Furthermore, previous studies often discuss T2I from a macro-perspective of implementation, policy, or instructional management, foregrounding how institutions regulate its use or integrate it into curricula (Ng et al., 2021; Chan, 2023). Most of these studies fail to examine how students negotiate their abilities with T2I under assessment pressure, while also ignoring the role of teachers as gatekeepers to critique and assessment. Taken together, more targeted frameworks and methods are therefore needed to explain how students, educators' judgment, and studio-based conditions dynamically interact in shaping T2I adoption.

### 2.3 Overall research design

**Figure 1:**
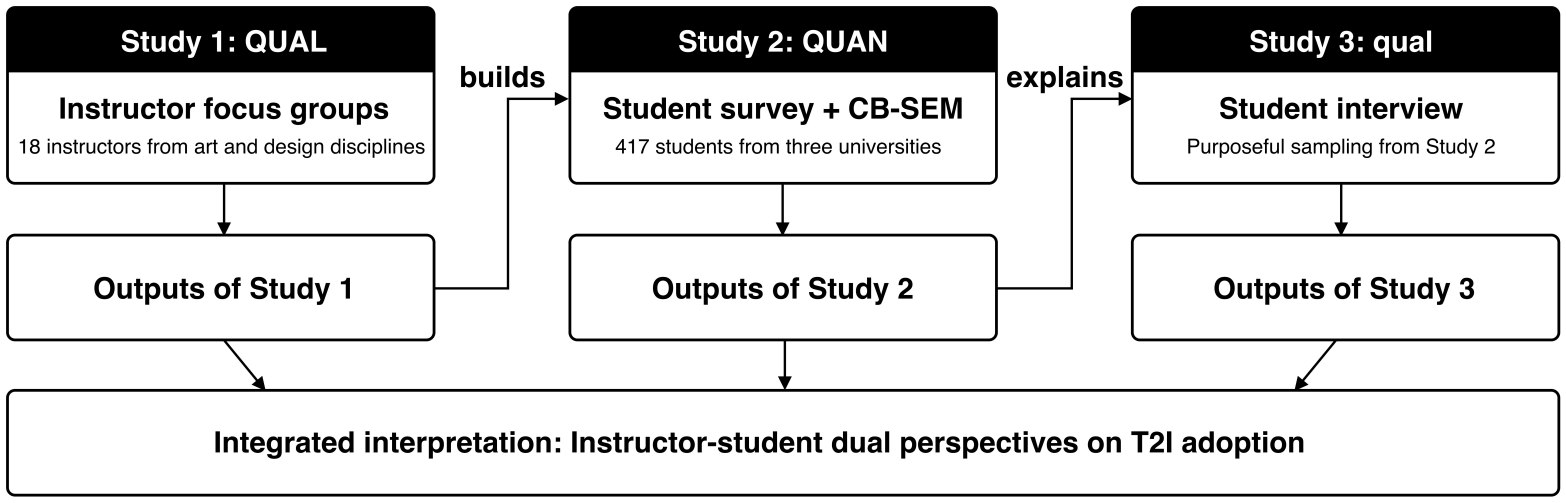
Overview of the three-phase sequential mixed-methods design.

To better capture the complex teaching and creative realities, this study draws on the exploratory sequential logic of mixed-methods research (Creswell and Plano Clark, 2018) and adopts a modified QUAL-QUAN-qual design with an explanatory qualitative follow-up phase (see Figure 1).

Specifically, Study 1 elicited context-specific themes from instructors to establish the baseline model framework and identify potential extension variables. Study 2 then translated these themes into an extended UTAUT model, testing the hypothesized relationships among the constructs within a student sample. Subsequently, Study 3 purposively selected interviewees based on anomalous quantitative profiles to provide a deeper understanding and interpretation of the statistical findings. Ultimately, this research design examines adoption behavior in creative education through a methodological logic of identifying, analyzing, and unpacking context-specific tensions in T2I GenAI adoption.

## 3 Study 1. Instructor focus groups

### 3.1 Participants and procedure

This exploratory qualitative phase employed a “building approach” (Shiyanbola et al., 2021), in which 18 instructors were recruited specifically from the art and design disciplines of a comprehensive university. To capture educators' perspectives on students' use of GenAI tools in terms of teaching processes and assessments, the focus groups were organized by different majors. Participants were divided equally into three groups (*n* = 6 per group): (1) visual communication design and digital media, (2) environmental design and product design, and (3) fine arts and illustration. Each session lasted approximately 50 min and was conducted in Chinese. All discussions were audio-recorded with the participants' informed consent. The semi-structured discussion guide was deductively informed by the baseline technology acceptance framework-covering topics such as perceived usefulness, ease of use, and instructional support-while remaining inductively open to studio-specific pedagogical processes, such as benchmark investigation, iterative concept development, critique and revision, and the optimization of design proposals.

### 3.2 Data analysis

The focus group data were analyzed using a hybrid deductive-inductive thematic approach following Braun and Clarke (2006). After verbatim transcription, coding proceeded iteratively, with initial codes informed by core technology acceptance constructs and refined through emergent domain-specific meanings. Constant comparison across the three groups led to the development of three themes that informed the subsequent survey design. NVivo 12 supported data management. Credibility was strengthened through second-coder review of 20% of the transcripts and member checking with a subset of participants.

### 3.3 Findings

Three themes emerged consistently across the focus groups.

Theme 1. Initial curiosity rarely translates into sustained engagement

Instructors observed that students' early enthusiasm for T2I tools is primarily driven by immediate experiential appeal and novelty, rather than a stable commitment to mastery. This initial attraction rapidly declines when students confront the procedural complexity and sustained effort required to refine AI outputs into pedagogically meaningful coursework.

Theme 2. Ease of use is exploited as a shortcut in academic tasks.

Because current T2I systems can quickly generate visually passable results, students frequently use them as instrumental shortcuts to bypass rigorous design processes. Consequently, their interactions often remain at a superficial conversational level, rather than evolving into highly intentional engagements rooted in algorithmic understanding or model fine-tuning. Paradoxically, because these effortless shortcuts sufficiently fulfill basic assignment requirements, they actively disincentivize students from pursuing deeper technical mastery. This dynamic complicates the traditional assumption that “ease of use” is inherently positive, highlighting a stark contrast between simple operational accessibility and meaningful creative control.

Theme 3. T2I reliance introduces a capability-training trade-off

Even among students capable of using advanced T2I prompting to meet basic assignment requirements, instructors noted their outputs were often technically adequate but aesthetically and conceptually hollow. Furthermore, educators expressed serious concerns that heavy reliance on AI displaces the time and effort needed to cultivate foundational disciplinary skills (e.g., hand drawing and original ideation). This reveals a profound tension between leveraging AI capabilities and the perceived risk of core professional skill degradation.

## 4 Study 2. Student survey

### 4.1 Quantitative theoretical framework and hypotheses

**Figure 2:**
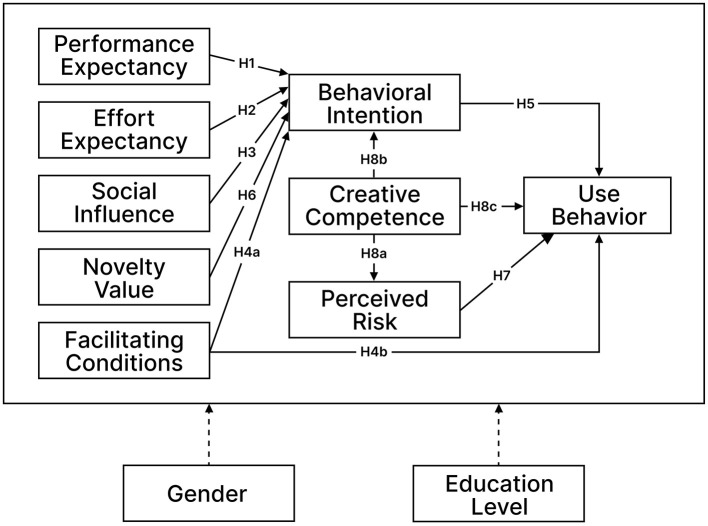
Research model.

Drawing on the qualitative insights from Study 1, instructors' observations aligned closely with the core dimensions of the UTAUT framework (performance expectancy, effort expectancy, social influence, and facilitating conditions). Accordingly, we used the four canonical variables as the baseline structure for the quantitative phase and then extended the model based on the findings from Study 1. Informed by prior conceptualizations (see Table 1), novelty value (NV) and perceived risk (PR) were included as key constructs, along with a new domain-specific construct, creative competence (CC). The resulting research model is presented in Figure 2.

**Table 1:** Qualitative building integration from Study 1 to Study 2.

Study 1 results	Keywords	Related constructs	Constructs focus
Theme 1	curiosity; declining persistence	novelty value; intention–use gap	novelty; exploratory appeal
Theme 2	shortcut use; shallow control	effort expectancy; creative competence	ease of use; prompting; revision; evaluation
Theme 3	originality skill loss; capability trade-off; evaluation pressure	creative competence; perceived risk	skill degradation; copyright; negative evaluation
Cross-theme implication	assessment norms; instructional goal; support conditions	performance expectancy; social influence; facilitating conditions	usefulness; social norms; support

#### 4.1.1 Core UTAUT predictors of T2I adoption

Although instructors expressed concerns regarding the negative teaching impacts of high usability, conventional technology adoption literature posits a different mechanism. Extensive UTAUT research—spanning foundational models (Venkatesh et al., 2003, 2012; Maruping et al., 2017; Dwivedi et al., 2019; Ursavaş, 2022), general GenAI adoption in higher education (Strzelecki, 2024; Xia and Chen, 2025), and specific T2I applications in creative domains (Ivanov et al., 2024; Shen et al., 2025; Huh et al., 2025)—consistently demonstrates that performance expectancy, effort expectancy, social influence, and facilitating conditions act as positive predictors of behavioral intention (BI) and actual use. Additionally, BI is established as a direct antecedent of use behavior (UB). Based on these precedents, we propose:

**H1:** *Performance expectancy (PE) has a positive influence on students' behavioral intention (BI) to use T2I GenAI tools*.
**H2:** *Effort expectancy (EE) has a positive influence on students' behavioral intention (BI) to use T2I GenAI tools*.
**H3:** *Social influence (SI) has a positive influence on students' behavioral intention (BI) to use T2I GenAI tools*.
**H4a:** *Facilitating conditions (FC) have a positive influence on students' behavioral intention (BI) to use T2I GenAI tools*.
**H4b:** *Facilitating conditions (FC) have a positive influence on students' use behavior (UB) of T2I GenAI tools*.
**H5:** *Behavioral intention (BI) has a positive influence on students' use behavior (UB) of T2I GenAI tools*.

#### 4.1.2 Novelty value (NV)

Although hedonic motivation serves as a standard driver in UTAUT2 (Venkatesh et al., 2012), novelty value (NV) offers a more precise lens for emerging GenAI systems (Xu et al., 2025). NV refers to the extent to which a product's originality and freshness stimulate user curiosity (Ma and Huo, 2023), and it has been shown to drive adoption to a degree that rivals or even exceeds that of social influence (Hmoud et al., 2024; Wang et al., 2022). We accordingly propose:

**H6:** *Novelty value (NV) has a positive influence on students' behavioral intention (BI) to use T2I GenAI tools*.

#### 4.1.3 Perceived risk (PR)

Perceived Risk (PR) is defined as the subjective expectation of potential loss or negative consequences associated with technology use to improve explanatory power in high-uncertainty contexts (Chen et al., 2024; Ivanov et al., 2024). In recent AI research, perceived risk is typically treated as multidimensional, and its effect on behavioral intention often appears to be indirect, particularly through trust (Jiang et al., 2023; Lai et al., 2024; Zhang et al., 2025). Many studies model PR as a negative antecedent of behavioral intention (Yakubu et al., 2025; Budhathoki et al., 2024) including ethical ambiguities around copyright and academic misconduct, and the fear of skill degradation through technological dependency (Kaya et al., 2024). Following the derivation of theme 3, we expected that more experienced students may be more sensitive to perceived risk than newcomers, and primarily as an execution-stage inhibitor. We therefore posit:

**H7:** *Perceived risk (PR) has a negative influence on students' use behavior (UB) of T2I GenAI tools*.

#### 4.1.4 Creative competence (CC)

To accurately capture different depths of engagement, a domain-specific measure of user capability is required. While user capability is typically operationalized as computer self-efficacy (CSE) (Compeau and Higgins, 1995) and AI competence (Long and Magerko, 2020), existing research predominantly conceptualizes these constructs in terms of functional and cognitive capabilities, often assuming a positive relationship between competence and technology use. This generalized construct is insufficient for evaluating T2I GenAI adoption in creative education, where competence also involves aesthetic judgment, authorship awareness, and demands more advanced control over semantic faithfulness and precise attribute realization (Chefer et al., 2023). Synthesizing these perspectives, we conceptualize Creative Competence (CC) as a domain-specific self-efficacy construct that captures a learner's perceived capability to execute prompt engineering for artistic purposes—specifically, the ability to translate conceptual ideas into effective prompts, steer outputs via advanced controls, iteratively revise generations, and critically evaluate their alignment with the intended creative vision.

Conceptually distinct from effort expectancy (EE), which reflects the perceived ease of learning and operating, CC represents the self-perceived capability to achieve creative outcomes through that system. According to self-efficacy theory, efficacy beliefs mitigate perceived threats in challenging tasks (Bandura, 1977). Consequently, we propose:

**H8a:** *Creative competence (CC) has a negative influence on students' perceived risk (PR) to use GenAI tools*.

Beyond risk perception, stronger efficacy beliefs promote motivation and persistence by raising individuals' confidence in successfully executing a target behavior (Bandura, 1977). We propose:

**H8b:** *Creative competence (CC) has a positive influence on students' behavioral intention (BI) to use GenAI tools*.

Competence beliefs also facilitate behavioral execution by enabling individuals to translate motivation into sustained action (Oppenlaender et al., 2025; Gist and Mitchell, 1992). We therefore propose:

**H8c:** *Creative competence (CC) has a positive influence on students' use behavior (UB) of GenAI tools*.

### 4.2 Method

#### 4.2.1 Measurement development

This study employed a quantitative survey design to validate the proposed research model. The measurement comprised two sections: (1) demographic information, including gender and educational level, which were later treated as potential moderating variables; and (2) items measuring the nine theoretical constructs. Seven predictor constructs and behavioral intention were assessed on a five-point Likert scale (1 = strongly disagree to 5 = strongly agree), while use behavior (UB) was operationalized on a five-point frequency scale (1 = never to 5 = always) through a frequency item asking how often students used T2I GenAI tools in current coursework. Because this item also functioned as a screening item, respondents who selected “Never” were not asked subsequent T2I-specific questions. In the final analytic sample, UB therefore represents self-reported use frequency among students with prior/current T2I experience rather than objective behavioral logs.

All items were adapted from established scales and further contextualized to the pedagogical settings of art and design education based on the findings of Study 1 (see Table 2). The questionnaire was conducted in Chinese. Before release, a pilot test with 10 students was conducted to verify content understanding and order adjustment. Respondents were informed that there were no right-or-wrong answers and that their responses would be kept confidential and used for research purposes only. Respondents were invited, but not required, to provide their contact information if they were willing to participate in a follow-up qualitative phase.

**Table 2:** Summary of measurement items.

Construct	Measurement items	Sources
PE	I find T2I GenAI tools useful for my studies. T2I GenAI tools help me complete design assignments/projects faster. T2I GenAI tools improve my creative productivity.	(Venkatesh et al., 2003)
EE	I can quickly master T2I GenAI skills (e.g., prompt writing). Using T2I GenAI tools feels effortless and not burdensome. I can use T2I GenAI to generate roughly the effects I want.	(Venkatesh et al., 2003)
SI	My instructors/supervisors encourage using T2I GenAI in art and design courses. My classmates/friends use T2I GenAI, which influences me. Renowned artists/design studios I follow use T2I GenAI, which influences me.	(Venkatesh et al., 2003)
FC	I have the hardware needed for T2I GenAI tools (e.g., a high-performance computer/GPU). I am able and willing to pay for T2I GenAI subscriptions. I can obtain necessary guidance and support from instructors/university when learning T2I GenAI.	(Venkatesh et al., 2003)
NV	Using T2I GenAI tools is a novel experience for me. T2I GenAI outputs often inspire my ideas. I find it enjoyable to explore different T2I GenAI models or platforms.	(Venkatesh et al., 2012) (Karjaluoto et al., 2019)
PR	I worry that relying on T2I GenAI will weaken my hand-drawing/original ability. I worry that using T2I GenAI in assignments/portfolios may cause copyright disputes or infringement. If instructors/judges know my work is AI-generated, I worry I will receive negative evaluations.	(Yakubu et al., 2025)
CC	I can accurately translate ideas into prompts to generate outputs. I can use advanced control techniques (e.g., LoRA, ControlNet) to steer outputs. When outputs are unsatisfactory, I know how to adjust prompts or parameters to improve them. I can evaluate T2I GenAI output quality and produce work that satisfies me.	(Gibreel and Arpaci, 2025) (Lo, 2023) (Lee and Palmer, 2025)
BI	I intend to continue using T2I GenAI tools in my future courses or creative projects. I plan to frequently integrate T2I GenAI tools into my daily learning workflow. Whenever possible (e.g., not prohibited), I will try to use T2I GenAI tools to assist my work.	(Venkatesh et al., 2003) (Foroughi et al., 2024)

#### 4.2.2 Sample and data collection

To ensure sample representativeness, questionnaires were administered to students from three purposively selected universities to provide variation within the Chinese art and design education context: an engineering-focused comprehensive university, a design-leading comprehensive university, and a specialized fine arts academy. Data collection was conducted between 25 January and 10 February 2026 via the online survey platform Wenjuanxing, with links distributed through social media groups. All participants provided informed consent before responding.

417 answers were returned. To ensure that respondents could meaningfully evaluate T2I-specific constructs, a screening item—“How often do you use T2I GenAI tools in current coursework?”—was placed at the start of the second section. The 17 respondents who selected “Never” were excluded. The remaining 400 responses were retained for analysis (valid retention rate: 95.9%). The final sample comprised 172 males (43.0%) and 228 females (57.0%); 288 were undergraduates (72.0%) and 112 were graduates (28.0%).

#### 4.2.3 Data analysis strategy

This study followed the two-step SEM procedure. In the first step, descriptive statistics and distributional assumptions were examined, and confirmatory factor analysis (CFA) was used to assess the measurement model across three criteria: internal consistency reliability, convergent validity, and discriminant validity. In the second step, the structural model was estimated to test the hypothesized paths; indirect effects were assessed via bootstrapping with 95% confidence intervals, with significance determined by whether the interval excluded zero. All analyses were conducted in Mplus and R using maximum likelihood estimation, and model fit was evaluated using χ^2^/df, CFI, TLI, and RMSEA.

Prior to multi-group comparisons, group-level reliability and convergent validity were inspected across gender and educational level to confirm baseline measurement comparability. Multi-group SEM was then used to explore potential heterogeneity in adoption mechanisms across demographic subgroups.

### 4.3 Results

Table 3 presents the descriptive statistics for the key constructs. Notably, Novelty Value (NV) and Behavioral Intention (BI) exhibited relatively high mean scores, reflecting strong student interest and adoption readiness. Conversely, Effort Expectancy (EE) recorded the lowest mean alongside a notably large variance, revealing highly dispersed user perceptions. Finally, skewness and kurtosis values for all items fell well within acceptable ranges, confirming the assumption of multivariate normality required for Maximum Likelihood Estimation (MLE) in subsequent SEM analyses.

**Table 3:** Descriptive statistics.

Variable	*N*	Min	Max	Mean	SD	Skewness	Kurtosis
NV	400	1	5	3.995	0.829	−0.923	0.921
CC	400	1	5	2.511	0.977	0.815	−0.009
EE	400	1	5	2.325	1.145	0.893	−0.182
FC	400	1	5	2.625	1.11	0.455	−0.725
PR	400	1	5	3.788	0.816	−0.786	0.496
BI	400	1	5	3.978	0.821	−0.994	0.913
PE	400	1	5	3.824	0.837	−0.537	−0.105
SI	400	1	5	3.818	0.831	−0.719	0.313
UB	400	1	4	2.835	1.132	−0.423	−1.252

#### 4.3.1 Measurement model evaluation

Table 4 and Appendix Table A1 detail the reliability, convergent validity, and item factor loadings of the measurement model. Most constructs demonstrated robust internal consistency and convergent validity, with Cronbach's alpha, composite reliability (CR), and average variance extracted (AVE) exceeding standard thresholds (0.70 and 0.50, respectively), although Perceived Risk (PR) exhibited comparatively weaker metrics. Therefore, PR was retained for theoretical reasons but interpreted cautiously in subsequent analyses. Discriminant validity was also confirmed: the square roots of AVEs satisfied the Fornell-Larcker criterion (Table 5), and most Heterotrait-Monotrait (HTMT) ratios (Appendix Table A2) remained below the liberal 0.90 threshold. Overall, the measurement model is deemed statistically adequate for subsequent structural equation modeling (SEM).

**Table 4:** Reliability and convergent validity.

Construct	Items	Cronbach'sα	CR	AVE	√AVE
NV	3	0.804	0.804	0.579	0.761
CC	4	0.828	0.828	0.546	0.739
PE	3	0.787	0.789	0.556	0.745
EE	3	0.899	0.899	0.747	0.865
FC	3	0.831	0.830	0.621	0.788
PR	3	0.628	0.673	0.426	0.653
SI	3	0.759	0.761	0.515	0.717
BI	3	0.785	0.788	0.554	0.745

**Table 5:** Fornell-Larcker criterion.

Construct	NV	CC	PE	EE	FC	PR	SI	BI
NV	(0.761)	−0.305	0.375	−0.078	−0.137	0.322	0.357	0.302
CC	−0.305	(0.739)	−0.192	0.702	0.576	−0.129	−0.110	−0.138
PE	0.375	−0.192	(0.745)	0.021	−0.021	0.344	0.314	0.280
EE	−0.078	0.702	0.021	(0.865)	0.566	−0.093	−0.067	−0.155
FC	−0.137	0.576	−0.021	0.566	(0.788)	−0.279	−0.120	−0.196
PR	0.322	−0.129	0.344	−0.093	−0.279	(0.653)	0.593	0.355
SI	0.357	−0.110	0.314	−0.067	−0.120	0.593	(0.717)	0.355
BI	0.302	−0.138	0.280	−0.155	−0.196	0.355	0.355	(0.745)

#### 4.3.2 Structural model and hypothesis testing

The structural model was estimated using maximum likelihood. As shown in Table 6, the absolute fit indices demonstrated acceptable overall fit (χ^2^/df = 3.176, RMSEA = 0.074 <0.08). Although the incremental fit indices (CFI = 0.873, TLI = 0.849) were marginally below the conventional 0.90 threshold, this is a well-documented and acceptable phenomenon in complex multidimensional models. Recent methodological studies and technology adoption literature indicate that incremental fit indices are systematically penalized by model size and complexity. Given that our extended UTAUT model incorporates 8 latent constructs, 25 observed indicators, and complex mediation paths, strict adherence to the 0.90 cutoff may lead to over-modification and theoretical distortion. Therefore, prioritizing theoretical integrity, no *ad hoc* modifications (e.g., freeing error covariances across constructs) were introduced solely to artificially inflate the fit indices. Supported by the adequate absolute fit (RMSEA), the model was deemed to possess acceptable explanatory power for the exploratory purposes of this study.

**Table 6:** Model fit indices for the structural model.

Index	χ^2^	*p* (χ^2^)	χ^2^/df	CFI	TLI	RMSEA	AIC	BIC
Value	870.263	<0.001	3.1761	0.873	0.849	0.074	27453.43	27864.56

The model explained 36.0% of the variance in behavioral intention (BI), 9.8% of the variance in self-reported use behavior (UB), and 3.7% of the variance in perceived risk (PR) (see Table 7). Although the model did not explain a large proportion of variance in UB and PR, PR still functioned as a significant downstream predictor of UB. This pattern indicates that the extended UTAUT framework was more effective in explaining students' cognitive readiness to use T2I GenAI than in predicting the frequency of enacted use.

**Table 7:** Coefficients of determination (R^2^) for endogenous constructs.

Type	Construct	*R* ^2^	*P*-Value
Observed	UB	0.098	0.01
Latent	PR	0.037	0.295
Latent	BI	0.36	0

**Figure 3:**
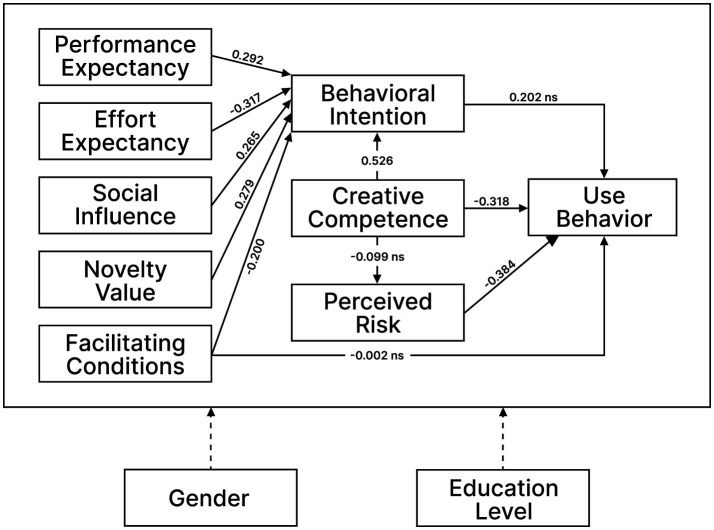
Results of model testing.

Table 8 and Figure 3 show the direct effects. PE, SI, NV, and CC positively predicted BI, supporting H1, H3, H6, and H8b. In contrast, EE and FC negatively predicted BI, contradicting H2 and H4a. For downstream outcomes, PR significantly and negatively predicted UB, supporting H7, whereas FC showed no significant direct effect on UB, leading to the rejection of H4b. The path from CC to PR was negative but statistically weak and is therefore interpreted as tentative rather than robust. Thus, H8a was interpreted as tentatively supported. BI showed only a weak and borderline association with UB, providing initial evidence that intention did not translate smoothly into self-reported use behavior in this context. H5 was partially supported. In addition, CC unexpectedly showed a significant negative direct effect on UB, contrary to H8c.

**Table 8:** Direct effects: path coefficients from the latent-variable SEM.

Path	Beta	SE	*p*	Boot95CI_Lo	Boot95CI_Hi
CC → BI	0.526	0.261	0.044	0.106	1.123
EE → BI	−0.317	0.153	0.038	−0.647	−0.063
FC → BI	−0.200	0.091	0.028	−0.409	−0.047
NV → BI	0.279	0.123	0.024	0.066	0.551
PE → BI	0.292	0.118	0.014	0.080	0.546
SI → BI	0.265	0.105	0.012	0.051	0.457
CC → PR	−0.099	0.054	0.066	−0.228	−0.013
BI → UB	0.202	0.104	0.051	−0.002	0.410
CC → UB	−0.318	0.121	0.008	−0.564	−0.091
FC → UB	−0.002	0.099	0.980	−0.187	0.202
PR → UB	−0.384	0.168	0.022	−0.741	−0.074

Bootstrapped indirect effects further clarified this pattern (Table 9). BI significantly mediated the effects of PE, SI, and NV on UB, while the negative effects of EE and FC on BI translated into negative indirect effects on UB. CC also showed a significant positive total indirect effect on UB through increased intention and reduced risk. However, this indirect support was offset by its negative direct effect on UB, indicating a countervailing structure in which creative competence simultaneously supports and constrains enactment.

**Table 9:** Indirect effects: path coefficients from the latent-variable SEM.

Path	Beta	SE	Boot 95% CI lower	Boot 95% CI upper
CC → (Total Indirect) → UB	0.144	0.083	0.025	0.359
CC → BI → UB	0.106	0.073	0.005	0.306
CC → PR → UB	0.038	0.027	0.003	0.116
EE → BI → UB	−0.064	0.043	−0.181	−0.004
FC → BI → UB	−0.040	0.027	−0.114	−0.003
NV → BI → UB	0.056	0.039	0.002	0.163
PE → BI → UB	0.059	0.036	0.006	0.160
SI → BI → UB	0.054	0.038	0.001	0.157

#### 4.3.3 Exploratory multi-group patterns by educational level and gender

To explore whether the proposed model operates differently across key demographic subgroups, we conducted a multi-group structural equation modeling (MG-SEM) analysis based on educational level and gender. These analyses are interpreted as strictly exploratory rather than confirmatory, given the limited subgroup sizes and the absence of formal measurement invariance testing. Overall, the model fit of the MG-SEM analyses by educational level and gender reached an acceptable standard.

Regarding educational level (see Table 10), subgroup differences were most evident in the CC-related paths. CC negatively predicted PR only among graduate students, whereas its negative direct association with UB appeared exclusively among undergraduates. Other antecedents of BI did not reach conventional significance in either subgroup, an observation suggesting that heterogeneity was concentrated less in intention formation than in the downstream role of competence.

**Table 10:** Exploratory multi-group path estimates by educational level.

Path	Group–undergraduate			Group–graduate		
	B	SE	*P*–value	B	SE	*P*–value
CC → BI	0.703	0.635	0.268	0.245	1.332	0.854
EE → BI	−0.430	0.385	0.264	−0.181	0.470	0.700
FC → BI	−0.209	0.143	0.144	−0.168	0.353	0.633
NV → BI	0.361	0.243	0.138	0.260	0.388	0.503
PE → BI	0.349	0.211	0.098	0.121	0.473	0.798
SI → BI	0.315	0.204	0.122	0.030	0.531	0.955
CC → PR	−0.041	0.053	0.441	−0.266	0.124	0.032
BI → UB	0.185	0.118	0.117	0.335	0.244	0.171
CC → UB	−0.367	0.147	0.013	−0.038	0.645	0.954
FC → UB	−0.115	0.124	0.356	0.230	0.280	0.411
PR → UB	−0.355	0.196	0.070	−0.136	1.136	0.905

As shown in Table 11, the gender-based subgroup estimates suggest different patterns in intention formation and enactment. In the female subgroup, SI and NV positively predicted BI, while BI positively predicted UB, and PR negatively predicted UB. Comparable focal paths were not statistically significant in the male subgroup. Nevertheless, given the larger standard errors and weaker reliability of PR in that cohort, these contrasts should be interpreted cautiously as exploratory subgroup patterns rather than definitive gender differences.

**Table 11:** Exploratory multi-group path estimates by gender.

Path	Group–female			Group–male		
	Beta	SE	*P*–value	Beta	SE	*P*–value
CC → BI	0.290	0.262	0.268	1.633	3.498	0.641
EE → BI	−0.141	0.154	0.360	−0.995	2.036	0.625
FC → BI	−0.154	0.130	0.234	−0.449	0.703	0.522
NV → BI	0.258	0.107	0.016	0.924	2.293	0.687
PE → BI	0.202	0.160	0.206	0.503	0.804	0.531
SI → BI	0.449	0.130	0.001	−0.232	1.154	0.841
CC → PR	−0.085	0.065	0.193	−0.126	0.090	0.163
BI → UB	0.374	0.123	0.002	−0.081	0.155	0.601
CC → UB	−0.334	0.187	0.074	−0.166	0.169	0.326
FC → UB	−0.081	0.164	0.621	0.019	0.130	0.880
PR → UB	−0.584	0.227	0.010	−0.056	0.253	0.825

### 4.4 Findings

The quantitative findings partly confirmed the tensions identified in Study 1. Students reported a relatively high level of novelty value, as NV had the highest mean score among the key constructs (M = 3.995, SD = 0.829). In the structural model, NV also significantly and positively predicted BI (β = 0.279, SE = 0.123, *p* = 0.024), indicating that the perceived novelty and exploratory appeal of T2I GenAI remained an important driver of students' intention to use these tools. Contrary to conventional UTAUT assumptions, EE (β = −0.317, SE = 0.153, *p* = 0.038) and FC (β = −0.200, SE = 0.091, *p* = 0.028) showed significant negative associations with BI. These results do not by themselves demonstrate shortcut-oriented use, but they are consistent with the concern raised in Study 1 that operational ease and available support may not necessarily lead to deeper engagement in studio-based creative learning. This interpretation was therefore treated as a pattern requiring qualitative explanation in Study 3. BI showed only a weak association with UB, and CC negatively predicted UB, corroborating the “novelty decay and execution gap” theme identified before. Together with the modest *R*^2^ for UB, this suggests that the extended UTAUT model captured intention formation more effectively than actual behavioral enactment, and that students' use of T2I tools in studio coursework is likely shaped by additional task-based, evaluative, and contextual conditions.

In addition, PR showed both low explained variance and internal measurement unevenness, with PR1 loading more weakly than PR2 and PR3. This pattern suggests that students may not conceptualize risk in T2I-supported studio learning as a single homogeneous construct. Rather, developmental risks may differ from external or evaluative risks. This finding connects directly to the instructors' concerns about tool reliance identified in Study 1 and provides a rationale for the follow-up interviews in Study 3.

## 5 Study 3. Student interviews

The model fit indices and unexplained variance in Study 2 suggest that certain contextual differences may remain uncaptured by the structural model, and the survey data alone cannot explain why students interpreted ease, support, competence, and risk in these ways, or why a similar background led to different usage. For this reason, to clarify these heterogeneous patterns across students with different disciplinary backgrounds, we employed qualitative interviews as part of an explanatory qualitative follow-up phase.

### 5.1 Participant selection

This phase adopted a purposeful sampling strategy and recruited participants directly from the original survey sample in Study 2. Based on survey responses corresponding to the anomalous model paths, three typical profiles were identified:

**A:** *less-experienced dependents*
**B:** *execution-stage retreaters*
**C:** *risk-sensitive restrainers*

This profile-based strategy was adopted to maximize explanatory value. Educational level and major were retained as background attributes during analysis, but were not used as strict sampling quotas. A total of 20 students were recruited, covering art and design majors represented in Study 1 and 2. An overview of the interview panel is provided in Appendix Table A3.

### 5.2 Data collection

The study employed individual semi-structured interviews in both online and face-to-face formats, each lasting approximately 45 min. All interviews were conducted with participants' consent and audio-recorded for transcription. The interview outline was developed from the findings of Study 2 to elucidate the unexpected and non-obvious quantitative patterns, focusing on four areas:

(1) *concrete cases in learning and coursework;*
(2) *the gap between initial intention and actual behavior;*
(3) *understanding of ease, control, and competence in T2I use;*
(4) *the nature of perceived risks, including internal, external, or other forms*.

### 5.3 Data analysis

The interview data were analyzed using Braun and Clarke's six-phase thematic analysis, which was explanatory in orientation: coding was guided by the results of Studies 1 and 2, while remaining open to unexpected meanings emerging from participants' narratives. Initial coding focused on concrete decisions to use / not use T2I in disciplinary learning and coursework. Related codes were then grouped into higher-order themes explaining the anomalous paths identified. Coding and theme refinement were discussed iteratively within the research team until a stable thematic structure was reached.

### 5.4 Findings of study 3

Students across three profiles commonly identified the baseline drivers of T2I adoption, including performance expectancy, social influence, and initial novelty value. However, the interviews revealed divergences in three critical transitions: from surface-level onboarding to in-depth mastery, from casual experimentation to formal studio execution, and from initial technological curiosity to the complex negotiation of perceived risks.

#### 5.4.1 Easy to start, hard to deepen

A recurring theme across the interviews was that T2I tools were easy to start but difficult to deepen. This pattern was especially evident among less experienced students. Although they recognized that advanced use required stronger technical knowledge, greater computing resources, and model-level control, they still engaged with T2I only superficially, using it to produce “good enough” outputs for studio assignments. This orientation can be understood as a form of task-threshold satisficing, and T2I use was not primarily aimed at expanding creative exploration or achieving stronger authorship, but at producing outputs that were visually complete enough to meet the minimum coursework threshold. In other words, students tended to stop refining prompts once the generated image reached the requirement, even though they knew the output lacked details, aesthetic depth, or conceptual development. As one student stated:

**P4:** *I know that more advanced functions such as LoRA training and parameter adjustment are very complex, and learning these technologies requires a lot of time and a better computer, but the free-tier version is enough to complete my course task at the present stage, so I have not made up my mind to further my study in T2I*.

#### 5.4.2 Why intention does not always become use

A second salient theme was the weak relationship between behavioral intention (BI) and use behavior (UB). Interview data suggest that this gap was closely related to differences in creative competence (CC), and also clarify why a single self-reported frequency item could not fully capture the quality, purpose, and stage of T2I use. Among participants with higher perceived CC, stronger capability was not necessarily associated with greater reliance on T2I. Instead, it often coincided with more critical judgment, allowing them to recognize technical bottlenecks such as unstable outputs, semantic inconsistency, and limited fine-grained control. These students tended to view T2I as a phased execution tool shaped by homogenized outputs and algorithmic aesthetics. This also reflects their awareness of the perceived creative ceiling of T2I systems: even after elaborate prompting, iterative refinement, or model training, the outputs could become more detailed and visually refined, but still appeared difficult to move beyond generic visual solutions or toward genuinely distinctive creative concepts. Therefore, compared with conventional technology adoption assumptions that treat higher competence as a facilitator of greater use, critical adoption more accurately captures this pattern of resisting over-reliance on technology while still using it selectively and purposefully. Consequently, they tended to confine T2I to early ideation stages and low-risk tasks, while avoiding its use in formal submissions, portfolios, and studio critiques.

**P8:** *The more familiar I became with it, the more I felt that it was just an execution tool. It does not really help with the creative or aesthetic part. When my well-trained models reached the algorithmic limit, their control over composition, space, and style also reached a ceiling. The results often became too generic and formulaic to meet my needs. So for complex tasks, I only use them at certain stages rather than rely on them for outcomes*.

For students with medium or lower perceived CC, two different patterns appeared. The first was moderate intention but relatively frequent use. For these participants, the attraction of output efficiency and the pressure of coursework deadlines functioned as situational catalysts, converting hesitation into pragmatic adoption and efficiency-driven compromise. The second pattern was moderate intention followed by low use after initial learning trials. These students found that the cost of learning and operating T2I tools was too high during the trial, and that producing original work directly was often faster and more manageable than investing substantial effort in mastering unfamiliar systems. Participant 11 explained this clearly:

**P11:** *I tried searching online for tutorials and even signing up for training courses to learn these tools. Honestly, for someone like me with limited computer skills, drawing by myself is faster than learning these tools from scratch. So I would rather wait until more user-friendly software appears in the future before learning them*.

#### 5.4.3 Perceived risk as an execution-stage deterrent

The weaker psychometric performance of risk in Study 2 was a distinct and stage-sensitive theme. The survey items captured several risk concerns, but the interviews suggested that students rarely described risk as a barrier to initial experimentation; instead, it became most salient when AI-assisted work moved from exploration to submission, critique, or formal evaluation. Students distinguished between evaluation-oriented risk, linked to submission legitimacy and external judgment, and development-oriented risk, linked to longer-term concerns about weakened originality or the loss of foundational skill, particularly the difficulty of integrating with T2I workflows. As one student explained:

**P20:** *Relying on T2I gradually crowds out my practice time, which concerns me. I once generated a compelling architectural rendering using AI, but I had no idea how to modify it in the rendering software—I simply lacked the underlying technical skills. So I have been returning to manual drafting to improve my basic software ability, rather than getting used to AI*.

Together, these descriptions help explain why perceived risk inhibits T2I use during the execution phase of studio-style learning, and why initial acceptance may not translate into sustained or final-stage use.

## 6 Discussion

### 6.1 Integrating findings

#### 6.1.1 When ease enables shortcut use

Instructor focus groups indicated that while students initially perceive T2I tools as novel and attractive, these tools may gradually become a shortcut for completing coursework. This interpretation aligns with (Ayaz and Yanartaş 2020) and Bervell et al. (2022), suggesting that early exploration and functional support are often overshadowed by habit and hedonic motivation. Consistent with this, the negative effects of EE and FC on BI observed in Study 2 contradicted conventional UTAUT assumptions; student interviews further clarified this through two contrasting usage patterns. On one hand, students who perceive these tools as difficult to access or navigate are nonetheless drawn to the immediacy of visual feedback. Under such productivity norms, the immediacy of T2I generation can transform operational ease into a shortcut for assignment completion. On the other hand, for students with deeper experience, the perceived ease and accessibility of T2I tools often render AI limitations more visible. This suggests a non-linear relationship: while novelty encourages initial experimentation, greater familiarity frequently exposes the constraints of AI-generated outputs, prompting more selective and restrained engagement.

#### 6.1.2 Risk at the execution stage

Perceived risk does not function as a stable individual trait that uniformly suppresses adoption; rather, it operates as a stage-sensitive filter—largely absent during early exploration, but sharply activated when AI-assisted work moves toward formal submission, critique, or external evaluation. This helps explain why PR was difficult to predict in the quantitative model, while still exerting a significant inhibitory effect on actual use. This interpretation aligns with studies showing that AI adoption in educational and creative contexts is shaped by assessment pressure, ethical ambiguity, authorship concerns, and perceived legitimacy (Huh et al., 2025; Luo, 2024; Lai et al., 2024). It is also supported by recent work on creative displacement anxiety and AI anxiety, which suggests that students' unease toward AI-assisted creation is multidimensional rather than uniformly inhibitory (Chung et al., 2025; Kai et al., 2026).

#### 6.1.3 The competence paradox

Creative competence demonstrates a dual mechanism of empowerment and filtering, working together with the diverse risks mentioned above to shape both intention and behavior. A similar expertise-based filtering pattern is also reflected in qualitative research on design practice, where more experienced designers are more cautious about over-reliance (Naqvi et al., 2025), but contrasts with Gibreel and Arpaci's (2025) assertion that prompt-engineering competence universally enhances continued intention. We argue that students with higher perceived CC exhibit greater restraint. Guided by the sociology of craftsmanship (specifically the value of material resistance) and Control-Value Theory, the threat of deskilling and the circumvention of creative difficulties diminishes students‘ sense of authorship, creative identity, and perceived system control, ultimately triggering frustration and resistance. However, this interpretation should not be read as a simple claim that competence directly reduces T2I use. Rather, based on our teaching experience, the instructor focus groups in Study 1, and the student interviews in Study 3, we interpret the negative CC → UB path as a context-specific pattern of selective restraint (though frequency-based UB cannot distinguish superficial from deliberate engagement). Therefore, the competence paradox should be understood not as a fixed causal boundary, but as a mixed-methods interpretation of how creative competence may simultaneously enhance students' practical capacity to use T2I tools and strengthen their judgment about when, whether, and to what extent such tools should be deployed.

### 6.2 Theoretical contributions

This study makes four theoretical contributions. First, it provides a contextualized extension of GenAI adoption by showing how T2I is used in studio-based art and design education. Second, this study makes a methodological contribution to GenAI acceptance in higher education through its three-phase sequential mixed-methods design. Third, this study introduces the domain-specific concept of creative competence and breaks the positive linear assumption in traditional technology acceptance models regarding computer self-efficacy. This provides a novel theoretical framework based on competence differentiation for the intention-behavior gap in human-computer collaborative creation within creative education research. Finally, in studio-based creative education, this study links perceived risk with technology adoption as a phased, conditional, and deliberative process. This provides a theoretical basis for future research on technology adoption in creative education.

### 6.3 Practical implications

The findings carry several practical implications for creative education. Firstly, because students differ in their interests, competencies, and perceived risks, we suggest that instructors identify different student profiles within specific courses and adopt differentiated teaching strategies with distinct developmental goals to better match and align pedagogical expectations with students' actual readiness. Secondly, institutions need to redefine AI literacy in design domains with a stage-sensitive approach to capability development and evaluation rather than treating “AI use” as a monolithic category and relying on uniform training or undifferentiated assessment. Finally, the recurring challenge emerging from the interviews was how students could integrate T2I with their own interests, capabilities, and goals, and incorporate it into a sustainable workflow. Accordingly, studio-based teaching and assessment should place greater emphasis on students' human-AI co-design processes. More broadly, the key educational question is no longer whether students can use AI tools, but which forms of judgment, creative capability, and responsibility remain essential in AI-assisted creative environments. Future curricula should therefore move beyond narrow tool training and instead cultivate students who can work with generative systems while exercising critical selection, aesthetic judgment, technical continuity, and ethical responsibility.

## 7 Conclusion

This study reconceptualizes T2I GenAI adoption in art and design education as a negotiated process where creative competence, identity, and perceived risks—rather than purely utilitarian factors—determine engagement. However, these findings should be interpreted in light of several limitations. First, the sample was drawn from art and design departments in a single national context and relatively well-resourced institutions, which should be interpreted as context-sensitive rather than universally generalizable. Second, the cross-sectional survey and retrospective interviews could not fully capture how students' perceptions and behaviors change across different stages of studio work. Future studies should adopt longitudinal or process-based designs to examine T2I adoption across ideation, execution, critique, and final submission. Third, the SEM model showed an acceptable but not optimal fit and explained limited variance in actual use behavior and perceived risk. Future research should validate the model across broader samples, test alternative estimators, and refine measurement tools for AI-assisted art and design education. In particular, a more rigorous multidimensional scale of perceived risk should be developed to capture developmental, evaluative, ethical, legal, and identity-related concerns in creative practice. Finally, the reliance on self-reported use behavior limits the precision of behavioral measurement. Future studies should incorporate behavioral logs, workflow records, or platform-generated usage data to better explain the intention-behavior gap in AI-supported creative education.

## Data Availability

The raw data supporting the conclusions of this article will be made available by the authors, without undue reservation.
