# Supplementary material for: The competence paradox: negotiating ease, risk, and creative identity in text-to-image generative AI use among art and design students
Source: Front Psychol. 2026 Jul 2;17:1858187. doi: 10.3389/fpsyg.2026.1858187 (PMC13374163; doi:10.3389/fpsyg.2026.1858187)
Supplement: Supplementary file 1 [file Supplementary_file_1.docx]

Supplementary Material

1. **Appendix**

Table A1. Factor Loadings of Observed Variables on Latent Constructs.

| **DV** | **IV** | **β (STDYX)** | **SE** | **p** | **Boot95CI_Lo** | **Boot95CI_Hi** |
| --- | --- | --- | --- | --- | --- | --- |
| BI | BI1 | 0.771 | 0.038 | 0.000 | 0.691 | 0.842 |
|  | BI2 | 0.776 | 0.037 | 0.000 | 0.695 | 0.843 |
|  | BI3 | 0.683 | 0.044 | 0.000 | 0.593 | 0.767 |
| CC | CC1 | 0.727 | 0.040 | 0.000 | 0.640 | 0.797 |
|  | CC2 | 0.740 | 0.037 | 0.000 | 0.659 | 0.804 |
|  | CC3 | 0.760 | 0.036 | 0.000 | 0.681 | 0.825 |
|  | CC4 | 0.729 | 0.039 | 0.000 | 0.643 | 0.795 |
| EE | EE1 | 0.865 | 0.019 | 0.000 | 0.821 | 0.897 |
|  | EE2 | 0.843 | 0.022 | 0.000 | 0.794 | 0.882 |
|  | EE3 | 0.885 | 0.016 | 0.000 | 0.848 | 0.913 |
| FC | FC1 | 0.831 | 0.025 | 0.000 | 0.781 | 0.874 |
|  | FC2 | 0.746 | 0.035 | 0.000 | 0.675 | 0.809 |
|  | FC3 | 0.784 | 0.038 | 0.000 | 0.703 | 0.850 |
| NV | NV1 | 0.796 | 0.037 | 0.000 | 0.712 | 0.860 |
|  | NV2 | 0.721 | 0.036 | 0.000 | 0.641 | 0.785 |
|  | NV3 | 0.763 | 0.039 | 0.000 | 0.679 | 0.831 |
| PE | PE1 | 0.747 | 0.034 | 0.000 | 0.672 | 0.807 |
|  | PE2 | 0.716 | 0.041 | 0.000 | 0.633 | 0.792 |
|  | PE3 | 0.772 | 0.034 | 0.000 | 0.702 | 0.838 |
| PR | PR1 | 0.375 | 0.061 | 0.000 | 0.250 | 0.490 |
|  | PR2 | 0.754 | 0.067 | 0.000 | 0.622 | 0.882 |
|  | PR3 | 0.754 | 0.067 | 0.000 | 0.633 | 0.898 |
| SI | SI1 | 0.688 | 0.047 | 0.000 | 0.584 | 0.771 |
|  | SI2 | 0.718 | 0.041 | 0.000 | 0.632 | 0.793 |
|  | SI3 | 0.745 | 0.042 | 0.000 | 0.653 | 0.819 |

Table A2. HTMT Values.

| **Construct** | **NV** | **CC** | **EE** | **FC** | **PR** | **BI** | **PE** | **SI** |
| --- | --- | --- | --- | --- | --- | --- | --- | --- |
| NV | — | 0.374 | 0.092 | 0.168 | 0.453 | 0.380 | 0.471 | 0.457 |
| CC | 0.374 | — | 0.814 | 0.694 | 0.179 | 0.171 | 0.238 | 0.139 |
| EE | 0.092 | 0.814 | — | 0.655 | 0.123 | 0.184 | 0.025 | 0.081 |
| FC | 0.168 | 0.694 | 0.655 | — | 0.386 | 0.243 | 0.026 | 0.151 |
| PR | 0.453 | 0.179 | 0.123 | 0.386 | — | 0.505 | 0.490 | 0.859 |
| BI | 0.380 | 0.171 | 0.184 | 0.243 | 0.505 | — | 0.357 | 0.461 |
| PE | 0.471 | 0.238 | 0.025 | 0.026 | 0.490 | 0.357 | — | 0.406 |
| SI | 0.457 | 0.139 | 0.081 | 0.151 | 0.859 | 0.461 | 0.406 | — |

Table A3. Demographic Information of Interviewees

| **ID** | **Major** | **Level** | **Profile** | **T2I Tools Used** |
| --- | --- | --- | --- | --- |
| P1 | Digital Media Art | BA | A (low CC, high UB) | Deevid AI, ERNIE Image |
| P2 | Environmental Design | BA | A (low CC, high UB) | ERNIE Image, Nano Banana |
| P3 | Fine Art | MA | A (low CC, high UB) | Deevid AI, DALL·E |
| P4 | Visual Communication Design | BA | A (low EE, high BI) | Deevid AI, ERNIE Image |
| P5 | Product Design | BA | A (low EE, high BI) | Deevid AI, ERNIE Image |
| P6 | Illustration | MA | A (low FC, high BI) | Deevid AI, Nano Banana |
| P7 | Visual Communication Design | BA | A (low FC, high BI) | Deevid AI, ERNIE Image |
| P8 | Fashion Design | MA | B (high CC, low UB) | Midjourney, Stable Diffusion |
| P9 | Environmental Design | BA | B (high CC, low UB) | Midjourney, ComfyUI |
| P10 | Visual Communication Design | MA | B (high CC, low UB) | Stable Diffusion, Midjourney |
| P11 | Digital Media Art | BA | B (high BI, low UB) | Midjourney, Leonardo.ai |
| P12 | Product Design | MA | B (high BI, low UB) | Midjourney, DALL·E |
| P13 | Fine Art | BA | B (high BI, low UB) | Leonardo.ai, Nano Banana |
| P14 | Environmental Design | MA | B (high BI, low UB) | Midjourney, Adobe Firefly |
| P15 | Product Design | MA | C (high CC, low UB) | Midjourney, Stable Diffusion |
| P16 | Digital Media Art | MA | C (high CC, low UB) | Stable Diffusion, ComfyUI |
| P17 | Fine Art | BA | C (high CC, low UB) | Midjourney, Adobe Firefly, DALL·E |
| P18 | Illustration | MA | C (high PR1 scorers) | Midjourney, Nano Banana |
| P19 | Visual Communication Design | MA | C (high PR1 scorers) | Adobe Firefly, Midjourney |
| P20 | Fashion Design | BA | C (high PR1 scorers) | Stable Diffusion, Midjourney |
